# Supplementary material for: Single cell analysis of kynurenine and System L amino acid transport in T cells
Source: Nat Commun. 2018 May 17;9:1981. doi: 10.1038/s41467-018-04366-7 (PMC5958064; doi:10.1038/s41467-018-04366-7)
Supplement: Supplementary file 1 — Supplementary Information [file 41467_2018_4366_MOESM1_ESM.pdf]

Supplementary Information

Single cell analysis of kynurenine and System L amino acid transport in T cells

Sinclair et al

Supplementary Figure 1

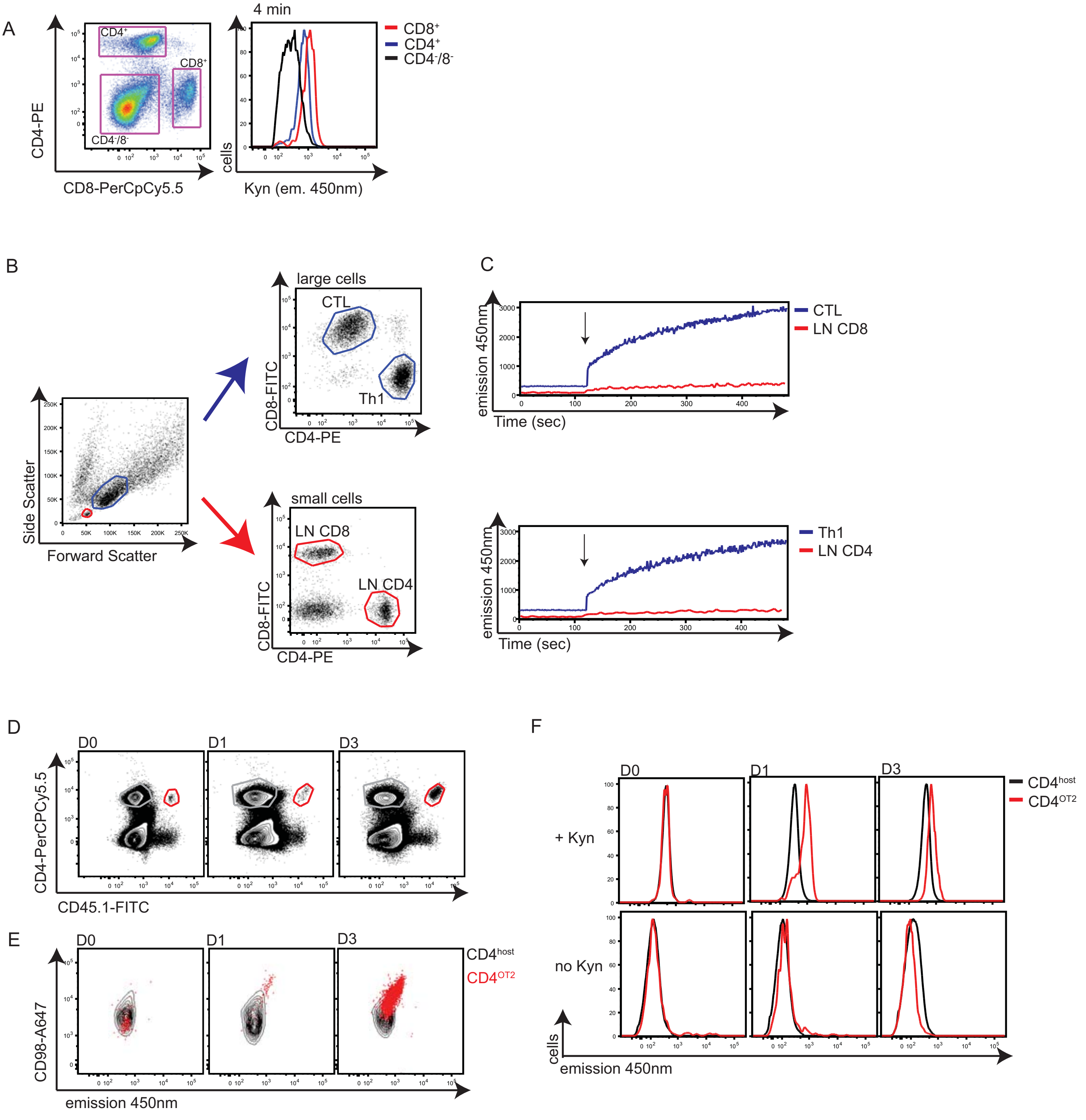

Supplementary Figure 1 Legend

a) Splenocytes were activated using anti-CD3/CD28 antibodies for 24h. The data show representative flow cytometry profiles of the gating strategy used to identify $CD4^+$, $CD8^+$ and $CD4^-/CD8^-$ populations (left panel). The overlaid histograms (right panel) show 450nm emission in $CD4^+$ or $CD8^+$ T cells or the $CD4^-/CD8^-$ gate after 4 mins (37°C) with kynurenine (200μM).

b,c) *In vitro* generated effecor CTL and TH1 were mixed with LN cells, prior to analysing live kynurenine uptake by flow cytometry. **b)** The gating strategy used to identify $CD4^+$ and $CD8^+$ LN and effector T cells. **c)** Kynurenine uptake in CTLs and LN $CD8^+$ T cells (top panel) or TH1 and LN $CD4^+$ T cells (bottom panel). Data acquired using 405nm excitation from the violet laser and band pass filter 450+/-50 on BD LSRII (Fortessa). Data acquired pre (120sec) and post (+600sec) addition of 200μM kynurenine (indicated by red arrow). The data are plotted as a geometric mean trace (FlowJo software).

d-f) OT2 (CD45.1) cells were adoptively transferred into WT CD45,2 hosts and immunised with NP-OVA/alum. The transferred OT2 cells were analysed on D0, D1 and D3 post-immunisation. **d)** The gating strategy to identify transferred OT2 ($CD4^+$, $CD45.1^+$) and host ($CD4^+$, $CD45.1^-$) T cells. **e)** the data show representative kynurenine uptake and CD98 staining on transferred OT2 $CD4^+$ T cells compared with host $CD4^+$ T cells after NP-OVA immunisation. **f)** The overlaid histograms show 450nm emission in OT2 $CD4^+$ or host $CD4^+$ T cells after 4 mins (37°C) with or without kynurenine (200μM).

Data shown are representative of 3 biological replicates.
